# HETEROSEXUAL AND SEXUALLY DIVERSE OLDER ADULTS’ LIFETIME EXPERIENCE OF IPV: FINDINGS FROM THE CLSA

**DOI:** 10.1093/geroni/igae098.2926

**Published:** 2024-12-31

**Authors:** Gloria Gutman, Mojgan Karbakhsh, Heather Stewart

**Affiliations:** Simon Fraser University, Vancouver, British Columbia, Canada; Simon Fraser University, Vancouver, British Columbia, Canada; Simon Fraser University, Vancouver, British Columbia, Canada

## Abstract

This study addresses knowledge gaps concerning prevalence and risk factors for intimate partner violence (IPV) among lesbian, gay and bisexual (LGB) compared to heterosexual Canadians aged 50+. Data derive from the Canadian Longitudinal Study on Aging (CLSA), a national cohort study with follow-up every 3 years for 20 years or until death. Participants who reported having ever been in an intimate partner relationship (n=11,727) who answered ‘yes’ to at least one item on the Composite Abuse Scale (Revised) – Short Form (CASR-SF) which was used to assess the prevalence of lifetime and past-year exposure to IPV and IPV types were included in this analysis. Main explanatory variables are sexual orientation and gender identity. Overall prevalence (exposure to any type) of IPV was 23.1% (16.6% among males; 29.1% among females) (p< 0.001). The lifetime prevalence rate was highest among LB women (35.8%), followed by GB men (29.3%), heterosexual women (29.0%) and heterosexual men (16.2%) (P< 0.001). The most common type of IPV was psychological (18%), with highest prevalence among LB women (27.5%) followed by heterosexual women (23.2%), GB men (20.4%) and heterosexual men (12.8%) (P < 0.001). The pattern of higher rates among sexually diverse woman and men then among their heterosexual age mates was also seen in lifetime experience of physical and sexual IPV. Recognition and prevention of IPV among sexually diverse older adults has been largely overlooked despite the fact that the IPV rates among same sex couples are reported to be similar or higher in comparison to opposite sex couples.

